# Collateral damages in the SARS-CoV-2 pandemia– two cases

**DOI:** 10.1186/s42466-020-00080-2

**Published:** 2020-11-11

**Authors:** Christian Urbanek, Jana Kötteritzsch, Wolfgang Zink, Armin J. Grau

**Affiliations:** 1 Department of Neurology, Klinikum der Stadt Ludwigshafen a. Rh. gGmbH, Bremserstr. 79, D-67063 Ludwigshafen, Germany; 2 Department of Anesthesiology and Intensive Care Medicine, Klinikum der Stadt Ludwigshafen a. Rh. gGmbH, Bremserstr. 79, Ludwigshafen, D-67063 Germany

## Abstract

**Background and aims:**

At present, “severe acute respiratory syndrome new coronavirus” (SARS-CoV-2) affects the whole world and has led to a pandemia with almost 2.000.000 infected patients in the mid of April 2020 (WHO). Thus, health care specialists primarily focus on therapy of corona disease 2019 (COVID-19) and a lot of effort has been undertaken to get more manpower on intensive care units. However, the number of patients with life threatening diseases other than COVID-19 like heart attacks or strokes has not changed at all. With a strong focus on COVID-19, there is a marked risk of diagnostic and therapeutic delays or misdiagnoses, potentially harming those patients. In this respect, we present two of those cases with the intent to improve the medical management of *“traditional*“ diseases in times of corona pandemia.

**Methods:**

We present two patients with diseases others than SARS-CoV-2. Both cases were treated in our institution, a tertiary care hospital in the Southwest of Germany.

**Results:**

One patient had a prolonged treatment on intensive care unit (ICU) because of heart failure following voluntary isolation because of fearing COVID-19 and subsequent shortage of medication. Another patient with hypothesis of COVID-19 of primary care physician because of fever and a history of skiing in a high risk region for SARS-CoV-2 was sent home for isolation. After disease progression, the patient presented in an external hospital with fever, pain in the right ear and tachypnea. Immediately, antibiotics were started at same day, but nevertheless, he developed a septic shock, leading to multi organ failure. In blood samples, bacteria *Streptococcus pyogenes* was found, without any signs of SARS-CoV-2-infection. Despite adequate antibiosis, the patient developed fixed pupils, brain edema and died because of massive brain edema.

**Conclusion:**

Focusing only on COVID-19 may lead to delayed diagnosis and therapy in patients with “traditional diseases”. These two cases impressively clarify medical challenges in times of SARS-CoV-2 pandemia. It is important to emphasize that physicians and health care professionals have not only to focus on COVID-19 and virus associated diseases, but also on adequate drug supply, intake and monitoring and differential diagnoses, respectively.

## Introduction

In December 2019, a cluster of pneumonia cases, causes by a recently identified ‘severe acute respiratory syndrome new coronavirus (SARS-CoV-2), occured in Wuhan, China [1]. Meanwhile, SARS-CoV-2 affects the entire world and has led to a pandemic with almost 2.000.000 infected patients in the mid of April 2020 [2]. Thus, health care specialists primarily focus on therapy of corona disease 2019 (COVID-19), and a lot of effort has been undertaken to get more manpower on intensive care units. However, the number of patients with life threatening diseases other than COVID-19 like heart attacks or strokes has not changed at all. With a strong focus on COVID-19, there is a marked risk of diagnostic and therapeutic delays or misdiagnoses, potentially harming those patients. In this respect, we present two of those cases with the intent to improve the medical management of *“traditional* “diseases in times of corona pandemia.

## Methods

We present two patients with diseases others than SARS-CoV-2. Both cases were treated in our institution, a tertiary care hospital in the Southwest of Germany. For case representation, we used medical reports and computer tomography imaging.

### Case 1

**Fig. 1:**
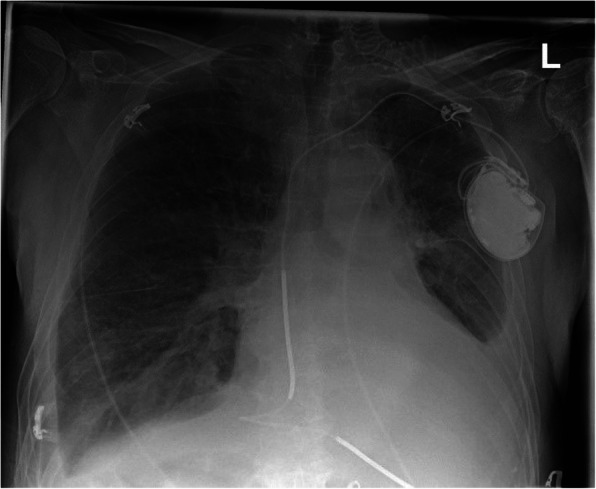
X-Ray 03/25/2020, left-sided pulmonal fluids

**Fig. 2:**
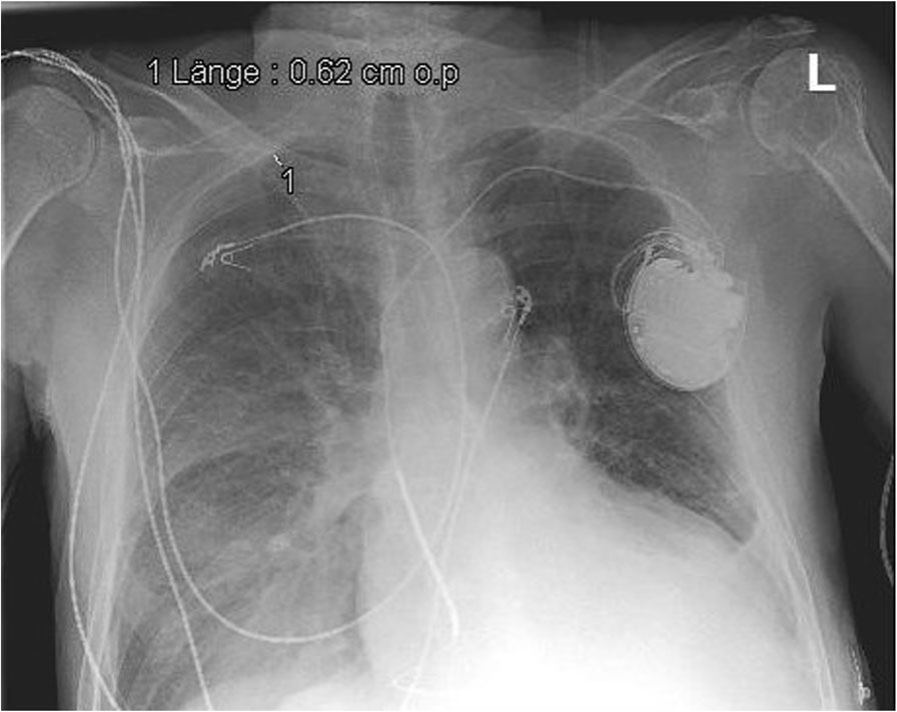
X-Ray 03/27/2020, lunge after draining 1.1 l pulmonal fluids

On March 25th 2020, a 70 years old patient suffering from heart failure (New York Heart Association III), atrial fibrillation, chronic renal failure (without need for dialysis) and with a history of stroke called the emergency service because of uncontrolled activation of his automatic implantable cardioverter-defibrillator (AICD). For fear of infection with SARS-CoV-2 and not for objective medical reasons, this patient decided to live in voluntary isolation. Consequently, he had no contact to his primary physician and because of an increasing shortage of his medication (especially: beta blocker, two diuretics and rivaroxaban), he stopped taking it 2 weeks before. Arriving in the emergency room, the patient was haemodynamically stable, but suffered from dyspnea. The AICD worked properly and had recorded six episodes of activation because of ventricular tachycardia within the last weeks. Furthermore, the patient did not complain about chest pain, and the ECG showed no signs of acute myocardial ischaemia. Subsequently, a chest X-ray was performed, with no signs obvious signs of cardiac congestion or pneumonic infiltrations, respectively. A few hours later, however, the dyspnea became worse, most probably due to progressive cardiac decompensation, and the patient had to be transferred to the ICU. Receiving 6 L of oxygen via the nasal route, he was clinically stable (arterial blood gas analysis: pO_2_ 68,6 mmHg, pCO_2_ 57,7 mmHg, pH 7339). Laboratory investigation showed no pathological findings at this time. However, within the next hours, dyspnea deteriorated, and the patients developed global respiratory failure, most probably due to progressive heart failure, with a left ventricular ejection fraction of approximately 35% (as assessed by transthoracic echocardiography). Thus, another chest X-Ray (Fig. 1) was done, now showing pleural effusions (which were subsequently drained) and progressive cardiac congestion. The dosage of diuretic (furosemid and spironolactone) was adjusted, and in the following hours, we managed to recompensate the patient (Fig. 2). However, patient developed fever, and because of an elevated white blood count, empirical antibiotic therapy was started using ampicillin/sulbactam and clarithromycin. Over the next 2 days, the need for oxygen decreased and the clinical status of the patient increasingly improved, and so it was possible to transfer him to the intermediate care unit. After 8 days in hospital, he was discharged in good health.

### Case 2

**Fig. 3:**
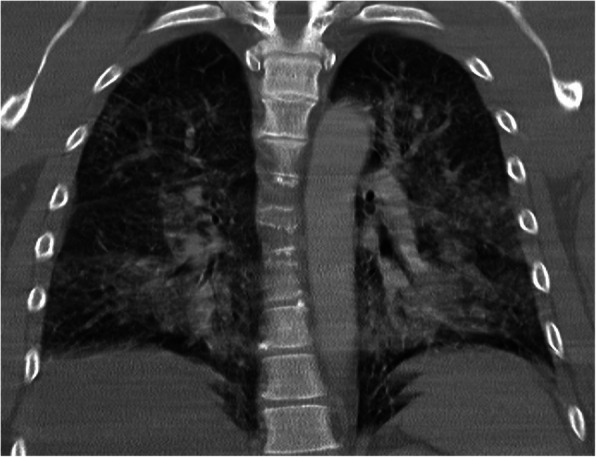
Thoracal Scan, 03/15/2020, pleural infiltrates, not characteristic for SARS-CoV-2

**Fig. 4:**
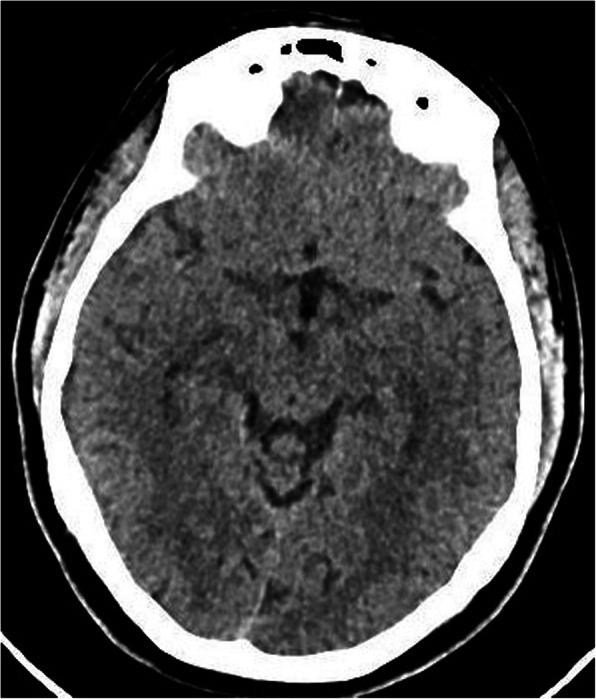
Brain Scan, 03/15/2020, no signs for brain edema

**Fig. 5:**
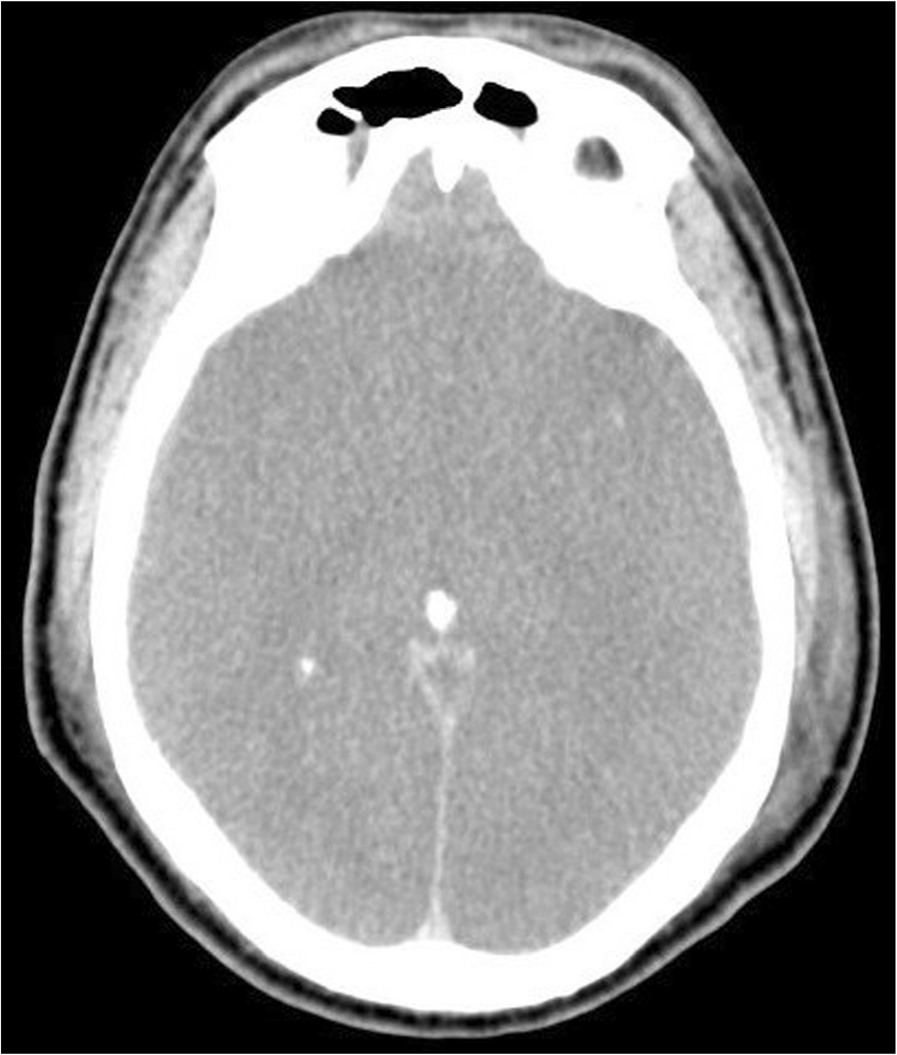
Brain Scan, 03/16/2020, swelling of whole brain

**Fig. 6:**
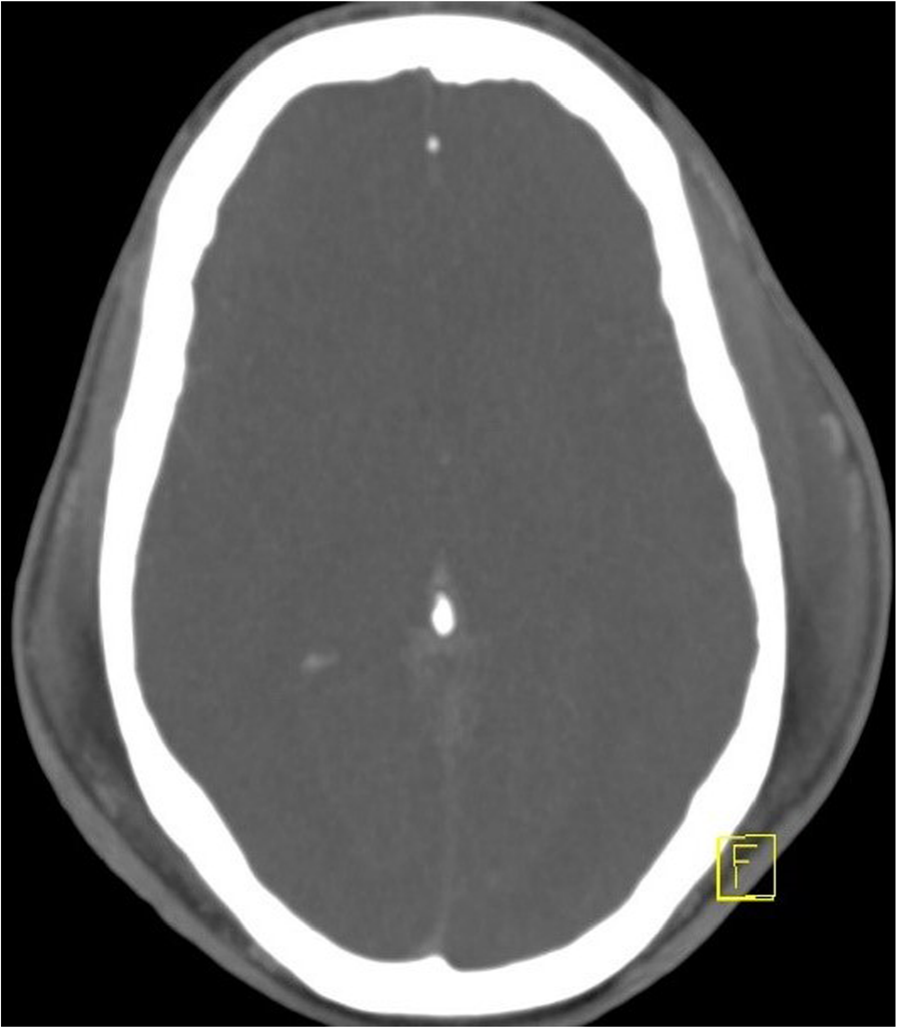
Brain Scan, 03/18/2020, evidence for brain dead

On March 13th 2020, a 39 years old patient developed right-sided aural pain. As he returned from a skiing holiday in a high risk region for COVID-19 2 days ago, his primary physician and ENT specialist by phone recommended testing for SARS-CoV-2, and dissuaded from a personal presentation in their ambulances. At this time point, pandemia of SARS-CoV-2 just started in Germany, and health care system has just started with building up testing units and fever ambulances. Thus, it was not possible for the patient to be tested in a timely manner. Following the advice of his primary physician, he stayed at home in voluntary isolation without haunting an emergency room. Two days later, aural pain tremendously deteriorated, with subfebril temperatures, dyspnea (without cough) and intermittent loss of consciousness. At the emergency department of an external hospital, he now presented with tachypnea (60/min.), tachycardia (105 bpm) and hypertension (191/83 mmHg). Suddenly, the patient became hypotensive and, subsequently, pulseless requiring cardio-pulmonary resuscitation, with a return of spontaneous circulation after 2 minutes. Meanwhile, the patient was intubated and artificially ventilated. In the following, the patient developed a prolonged tonic-clonic seizures, which were successfully treated with benzodiazepines. Laboratory investigations showed an elevated white blood count (17.0 10^3^/μl), highly elevated levels of CRP (319 mg/dl) as well as of procalcitonin (96.4 μl/l) in means of a severe sepsis. As signs of a dissiminated intravascular coagulation, D-dimers and International Normalized Ratio (INR) were also increased. Antibiotic therapy with ciprofloxacin and piperacillin/tazobactam was immediately started, and volume resuscitation as well as and intravenous vassopressors were established. A ear, nose and throat (ENT) specialist physicians diagnosed a severe otitis media, antibiotic therapy was escalated to meropenem. In addition, low-dose hydrocortisone was started due to persisting haemodynamic instability. At the same day, acute renal failure became evident, requiring immediate renal replacement therapy, and leading to a change in anticonvulsive drugs from valproate acid to lacosamid. Computertomography of the brain, chest and abdomen was performed, and because of pleural infiltrates (and with regard to the patient’s history), COVID-19 was discussed as possible diagnosis. However, radiological findings were not characteristic for this disease (Fig. 3 and Fig. 4). Furthermore, CT scans did not reveal signs of cerebral or abdominal infection, respectively. Because of an increasing need for vasopressors and progression of the sepsis the next day, the patient was transferred to our hospital. After arriving at the intensive care unit of our hospital, fixed and dilated pupils were assessed. Immediately, another CT scan of the brain was performed which showed swelling of the whole brain (Fig. 5). We hypothesized that a right-sided otitis media was the septic focus, leading to bacterial meningoencephalitis and subsequent loss of consciousness. Meanwhile, the screening for influenza and SARS-CoV-2, respectively, were negative. In the following, antibiotic therapy was escalated to intravenous ceftriaxone (4 g/d) according to current guidelines, and brain edema was treated. Because of an obviously elevated intracranial pressure, lumbar liquor puncture was contraindicated. In blood cultures, *Streptococcus pyogenes* was found, and antibiotics were adjusted to penicillin G and clindamycin. However, despite all therapeutic efforts, patient was brain dead 2 days later (Fig. 6).

## Results

We present two patients severe life-threatening diseases others than COVID-19 whose diagnoses and treatments were markedly affected by the current pandemia. One patient had a prolonged treatment on intensive care unit (ICU) because of heart failure following voluntary isolation because of fearing COVID-19 and subsequent shortage of medication. Another patient with hypothesis of COVID-19 of primary care physician because of fever and a history of skiing in a high risk region for SARS-CoV-2 was sent home for isolation. After disease progression and within few days, the patient developed fixed pupils and brain edema and died because of massive brain edema. However, although both patients were not infected with SARS-CoV-2, both disease progression were strongly associated with SARS-CoV-2 pandemia**.**

## Discussion

We present two patients severe life-threatening diseases others than COVID-19 whose diagnoses and treatments were markedly affected by the current pandemia. Without doubt, it is profoundly necessary and reasonable that health care systems are focusing and preparing on the management of patients with COVID-19. However, prevalence and incidence of non-viral diseases like heart failure, pneumonia and stroke have not changed at all.

Based on two clinical cases, it is our intention to highlight the apparently trivial fact that “normal“ diseases and “normal” patients still exist in times of COVID-19, and still must be recognized and treated. Voluntary isolation due to a fear of infection and thus, postponed visits at primary care physicians may have deleterious consequences like the discontinuation of necessary medication as seen in our case. Furthermore, patients may trivialize their symptoms just in order not to visit physicians or not to be admitted to a hospital. In consequence, adequate diagnosis as well as the subsequent initiation of a life-saving therapy may be delayed or even prevented. In our opinion, it is an important fact to inform and educate all patients that despite COVID-19, medical help and support is still available, and that medical practices and emergency rooms are safe shelters of help.

In conclusion, these cases and adequate management of No-COVID-19-patients represents a tremendous challenge for all health care professionals in these days.

## Data Availability

Authors state that all data are available.

## Abbreviations

AICD: Automatic implantable cardioverter-defibrillator
COVID-19: Corona disease 2019
ENT: Ear, nose and throat
INR: International Normalized Ratio
SARS-CoV-2: Severe acute respiratory syndrome new coronavirus
WHO: World Health Organisation
